# The Mediating Role of Selfitis in the Associations between Self-Esteem, Problematic Social Media Use, Problematic Smartphone Use, Body-Self Appearance, and Psychological Distress among Young Ghanaian Adults

**DOI:** 10.3390/healthcare10122500

**Published:** 2022-12-10

**Authors:** Derek Oppong, Emma Sethina Adjaottor, Frimpong-Manso Addo, Worlali Nyaledzigbor, Amma Serwaa Ofori-Amanfo, Hsin-Pao Chen, Daniel Kwasi Ahorsu

**Affiliations:** 1 Department of Psychology and Education, College of Educational Studies, University of Cape Coast, Cape Coast CC145, Ghana; 2 Department of Behavioural Sciences, Kwame Nkrumah University of Science and Technology, Kumasi AK-4944, Ghana; 3 Department of Social Sciences, Faculty of Arts and Social Sciences, Central University, Greater Accra GN0370, Ghana; 4 Division of Colon and Rectal Surgery, Department of Surgery, E-DA Hospital, Kaohsiung 824, Taiwan; 5 School of Medicine, College of Medicine, I-Shou University, Kaohsiung 824, Taiwan; 6 Department of Rehabilitation Sciences, Faculty of Health & Social Sciences, The Hong Kong Polytechnic University, 11 Yuk Choi Rd Hung Hom, Hong Kong 999077, China; 7 Mental Health Research Centre, The Hong Kong Polytechnic University, 11 Yuk Choi Rd Hung Hom, Hong Kong 999077, China

**Keywords:** selfitis, selfie, young adult, depression, anxiety, stress, problematic smartphone use, problematic social media use, coping skills, body-self appearance

## Abstract

Selfie-related activities have become pervasive to the point that they may affect the mental health of people who engage in them. To ascertain this mechanism, this study examined the mediating role of selfitis in the associations between self-esteem, problematic social media use, problematic smartphone use, body-self appearance, and psychological distress among young Ghanaian adults. A total of 651 participants completed a questionnaire with measures on self-esteem, body-self appearance, problematic social media use, problematic smartphone use, depression, anxiety, stress, coping skills, and selfitis. There were direct associations between all the variables except between self-esteem and selfitis. In addition, selfitis mediated the associations between problematic social media use, problematic smartphone use, body-self appearance, and psychological distress except between self-esteem and psychological distress. These findings suggest that selfitis can serve as a pathway by which people who overly engage in problematic social media use, problematic smartphone use, and have poor body-self appearance may experience psychological distress. Hence, there is a need for health communicators, school authorities, and opinion leaders to educate young adults on the consequences of the problematic use of technology, especially for selfitis behaviour. Future studies can examine the factors that predict selfitis behaviour among adults.

## 1. Introduction

Technological advancement has transformed our way of life in areas such as health [1,2,3], entertainment [1,4], and commerce [1,5,6]. Although the use of technology has several advantages, its misuse can be detrimental to the health, security, and finances of the user [7,8,9,10,11,12,13,14]. One of the trendiest uses of technology as a communication, entertainment, and economic gadget is selfie-taking [15,16]. A selfie is a picture taken of oneself for personal use or to post on social media usually using a smartphone [17]. The incidence of selfie-taking is high, especially among young adults with a prevalence of dangerous selfies being 8.74% [18] and overall selfie addiction being 13.88% (22.5% for females and 11.4% for males) [19]. 

Obsessive–compulsive engagement in selfie-related activities may be termed selfitis [20,21]. Beginning as a hoax [22], selfitis was defined as “obsessive–compulsive desire to take photos of oneself and post them on social media as a way to make up for a lack of self-esteem and to fill a gap in intimacy” (p. 1) [21,22]. Hence, by this definition, selfitis seems to be associated with self-esteem, social media, and cameras or camera phones. This further suggests that there may be more factors involved with selfitis such as environment enhancement, social competition, attention seeking, mood modification, self-confidence, and subjective conformity [21]. Although not a mental disorder yet, overly engaging in selfitis may pose a danger and/or interfere with a person’s school, work, and/or psychosocial functions and health [18,20,21,23]. The reasons for taking selfies have been discussed in the literature [21,24,25,26]. Even though it has been clearly stated that selfitis is not an addiction or a mental disorder, there seems to be a strong implied presence of “selfie addiction” [21,23]. That is, there is a suggestive aspect of addiction attached to selfitis taking into consideration the obsessive–compulsive characteristic and the need to fill gaps in self-esteem and intimacy. The addiction model (behavioural addiction) suggests that there should be salience (the most important everyday activity), conflict (interpersonal and intrapsychic conflict related to the activity), tolerance (increasing the levels of activity to achieve the previous effect), withdrawal symptoms (unpleasant effect after stopping an activity), mood modification (feelings or experience felt during an activity), and relapse or reinstatement (revisiting earlier undesirable activity after a period of control or abstinence) [23,27] which can be used to explain selfitis in its extreme form. Interestingly, self-esteem and body-self appearance are closely associated with selfitis. The addiction model has also been used to explain problematic social media use and problematic smartphone use [7,8]. The emergence of social media platforms and smartphones has intensified the taking of selfies [21,24,25,26]. This has led people to undertake dangerous poses even at a higher risk to themselves and/or others around them in order to experience the subjective euphoria or fame associated with posting them on social media platforms [28,29]. 

Social media has several advantages but like any technology, misusing it may be problematic to the individual. That is, the tendency to be addicted to it is high when one overuses it and such addiction can be associated with other mental health conditions [30,31]. For instance, some studies have reported an association between problematic social media use and selfitis [32]. Other studies have also reported an association between problematic social media use and other mental health conditions such as depression, anxiety, and self-esteem [7,10,33,34]. In terms of the direction of influence, problematic social media use can directly influence psychological distress (e.g., depression, anxiety, stress, and/or other mental disorders) [35,36,37,38]. 

Closely related to problematic social media use is problematic smartphone use. A smartphone is handy, sophisticated, and can be used for multiple tasks such as surfing the net or social media sites, taking pictures or videos, and/or receiving and making calls. This makes it easier for it to be abused and for people to become addicted to it. Problematic smartphone use, which is defined as an excessive dependence on a smartphone to the point that it causes significant challenges/interference with the person’s occupation and health, is significantly associated with mental health challenges. Some of these health problems include depression [7], anxiety [7], low self-esteem [39], problematic social media use [7,8,31], body dissatisfaction [40], and selfitis [24]. Problematic smartphone use has been found to directly influence psychological distress [36,37,41,42]. 

Additionally, the taking of selfies relies heavily on body-self appearance—how a person sees, thinks, and feels about himself or herself [43]. That is, a selfie basically involves taking a picture of oneself and posting it on social media. It could be assumed that the one taking the selfie will feel good and probably proud of his or her body-self appearance. Therefore, people may go to extreme lengths to look good in order to be liked, cheered, loved, and obtain more followers. However, not all selfie posts receive positive feedback. Body shaming, online/social media bullying, and ridicule are some of the negative responses that some have received [44,45]. Hence, there are associations between feedback, body satisfaction and selfie posting [19,29,45,46,47,48]. It is also worth noting that body-self appearance (e.g., body image) can directly influence psychological distress [49,50]. Closely related to selfie posting, feedback, and body satisfaction is self-esteem [29,45,46]. Self-esteem, defined as “reverence for self” and/or self-worth [51,52], has been found to directly influence psychological distress [53,54,55]. All these variables are also associated with psychological distress [29,45,46]. The research findings above, notwithstanding, one study found no relationship between selfies, self-esteem, and body image [56]. 

Several studies have examined selfitis as a predictor in mediation analysis [46,48,57], but, so far, only one known study has examined selfitis as a mediator [32]. Selfitis has been observed as a mediator between personality and problematic social media use [32]. This indicates that selfitis may also play mediating roles in other associations. This formed the basis of the present study as it expands on the mediating role of selfitis. Therefore, this paper examines the mediating role of selfitis in the associations between self-esteem, problematic social media use, problematic smartphone use, body-self appearance, and psychological distress. Specifically, it examines (1) whether there are significant associations between the variables of interest and (2) whether selfitis mediates the association between these variables.

## 2. Materials and Methods

### 2.1. Setting, Design, and Participants

This cross-sectional survey design study used Kwame Nkrumah University of Science and Technology (KNUST), a university in Ghana, as its setting. The data collection started with obtaining ethical approval from the research ethics committee of KNUST (Ref: CHRPE/AP/335/22). After ethical approval, we sought permission from several lecturers of KNUST in order to use their class and students for the data collection. After obtaining their permission, a date was set for the data collection. On the set date, we informed the students about the nature of the study and explained that their participation was voluntary. Students who were available and willing to participate signed the informed consent form after which they were handed the questionnaire. That is, the inclusion criteria for this study included being a university student, male or female, and being 18 years and above. The sample size formula N ≥ 50 + 8m (where m and N are the number of predictors and the sample size, respectively) was used as the rule of thumb for estimating the sample size for multiple correlations [58,59]. We thanked all the participants and gave each of them a KNUST-embossed pen as a token of our appreciation. The data collection procedures followed ethical principles and standards that conform with the Helsinki Declaration. The data were collected between May and June 2022. 

### 2.2. Measures

#### 2.2.1. Sociodemographic Characteristics

This section solicited participants’ sociodemographic data, including age, sex, religion, and marital status (see Table 1 in the results section for further details). 

#### 2.2.2. Single-Item Self-Esteem Scale (SISE) 

The SISE measured self-esteem using a single item “I have high self-esteem”. It is rated on a 5-point Likert scale (1 = not very true of me to 5 = very true of me) and has been shown to have high correlations with the Rosenberg self-esteem scale and a high test-retest reliability after four years (*r_tt_* = 0.75) [60]. A higher score indicates higher levels of self-esteem. 

#### 2.2.3. Multidimensional Body-Self Relations Questionnaire–Appearance Scales (MBSRQ-AS)

The MBSRQ-AS is a self-report questionnaire which consists of 34 items and five subscales (e.g., appearance evaluation scale, body areas satisfaction scale, and appearance orientation scale) used to assess different appearance-related aspects of body image. However, this study used only the subscales of 28 items. Its items are rated on a 5-point Likert scale, but the subscales have different response labelling. For example, the appearance evaluation scale and appearance orientation scale have their responses rated as 1 = definitely disagree to 5 = definitely agree while the body areas satisfaction scale is rated as 1 = very dissatisfied to 5 = very satisfied. The sample items for the appearance evaluation scale are “My body is sexually appealing” and “I like my looks just the way they are”. The sample items for the body areas satisfaction scale are “Face (facial features, complexion)” and “Lower torso (buttocks, hips, thighs, legs)”. The sample items for the appearance orientation scale are “Before going out in public, I always notice how I look” and “I am careful to buy clothes that will make me look my best”. All the participants’ responses (i.e., either for the general scale or subscales) are averaged together to obtain a mean score after reversing contra-indicative (or negatively worded) items. Therefore, higher mean scores indicate higher levels of body-self appearance and its subscales (i.e., appearance evaluation, body areas satisfaction, and appearance orientation). The scale and its subscales showed acceptable psychometric properties [61,62,63]. In this study, the Cronbach’s alpha coefficient observed for the general scale is 0.837.

#### 2.2.4. Selfitis Behaviour Scale (SBS) 

The SBS is a 20-item scale that assesses six domains of selfitis (i.e., environment enhancement, social competition, attention seeking, mood modification, self-confidence, and subjective conformity). Its items are rated using a five-point Likert scale (1 = strongly agree and 5 = strongly disagree). The sample items for environment enhancement (i.e., having an enjoyable environment, feeling good, and taking selfies for memories) are “Taking selfies gives me a good feeling to better enjoy my environment” and “I am able to express myself more in my environment through selfies”. The sample items for social competition (i.e., employing creative tactics to serve one’s socially competitive needs) are “Sharing my selfies creates healthy competition with my friends and colleagues” and “Taking different selfie poses helps increase my social status”. The sample items for attention seeking (i.e., one’s ability to gain more attention than others) are “I gain enormous attention by sharing my selfies on social media” and “I feel more popular when I post my selfies on social media”. The sample items for mood modification (i.e., feelings or experiences felt during an activity) are “I am able to reduce my stress level by taking selfies” and “Taking more selfies improves my mood and makes me feel happy”. The sample items for self-confidence (i.e., trusting oneself and abilities) are “I feel confident when I take a selfie” and “I become more positive about myself when I take selfies”. The sample items for subjective conformity (i.e., one’s obligation to follow social norms) are “I gain more acceptance among my peer group when I take a selfie and share it on social media” and “I become a strong member of my peer group through selfie postings”. The participants’ responses are summed to obtain an overall score and a higher score indicates higher levels of selfitis behaviour. It has acceptable internal consistency [21]. The Cronbach’s alpha coefficient observed for this study is 0.932.

#### 2.2.5. Depression Anxiety Stress Scale (DASS-21) 

The DASS-21 is a 21-item self-report scale that assesses depression, anxiety, and stress symptoms. Its items are rated on a four-point scale from 0 (“does not apply to me at all”) to 3 (“applies to me very much or most of the time”). The sample items are “I found it hard to wind down” and “I tended to overreact to situations”. Scores for each subscale are obtained by summing the responses of the items of that subscale. Higher scores indicate higher levels of depression, anxiety, and stress in the past week. It has acceptable internal consistency [64]. In this study, the Cronbach’s alpha coefficients observed for depression, anxiety, and stress are 0.809, 0.739, and 0.752, respectively. 

#### 2.2.6. Bergen Social Media Addiction Scale (BSMAS) 

The BSMAS is a six-item scale that assesses social media addiction. The six items examine the experience of using social media over the past year and the participants respond to it using a five-point Likert-type scale ranging between 1 (very rarely) and 5 (very often). The sample items are “Felt an urge to use social media more and more” and “Used social media to forget about personal problems”. A higher score in the BSMAS indicates a greater likelihood of being at risk of developing social media addiction. It has acceptable psychometric properties [65,66]. The Cronbach’s alpha coefficient observed for this study is 0.653.

#### 2.2.7. Smartphone Application-Based Addiction Scale (SABAS)

The SABAS is a six-item scale that assesses smartphone use addiction using a six-point Likert-type scale that ranges between 1 (strongly disagree) and 6 (strongly agree). The sample items are “My smartphone use results in conflicts” and “Preoccupying myself with my smartphone is a way of changing my mood”. A higher score in the SABAS indicates a greater likelihood of being at risk of developing an addiction to smartphone use. It has acceptable psychometric properties [67]. The Cronbach’s alpha coefficient observed for this study is 0.660.

### 2.3. Data Analysis 

**Figure 1:**
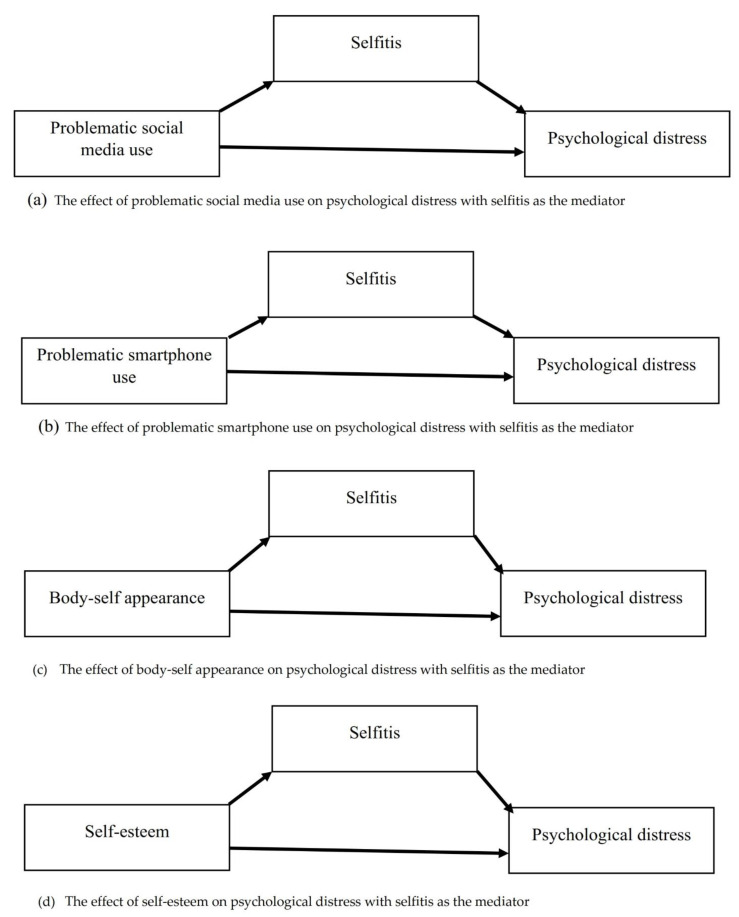
The hypothesized mediating role of selfitis in the associations between self-esteem, problematic social media use, problematic smartphone use, body-self appearance, and psychological distress among young Ghanaian adults.

Information on the demographic characteristics of participants was presented using means and standard deviations (M ± SD), and frequencies with their percentages. In addition, Pearson r was used to examine the relationships between selfitis, self-esteem, problematic social media use, problematic smartphone use, body-self appearance, and psychological distress. After that, four mediation analyses were performed using Hayes’ PROCESS macro version 4.1 for SPSS [68]. The mediation analysis used model 4 and 5000 bootstrapping resamples. The predictor variables were problematic social media use, problematic smartphone use, body-self appearance, and self-esteem. The mediating variable was selfitis and the outcome variable was psychological distress (see Figure 1). The level of significance was set at 0.05. All these statistical analyses were conducted using SPSS version 23 software for Microsoft Windows (Armonk, NY, USA: IBM Corp.).

## 3. Results

Table 1 displays the sociodemographic characteristics of the participants (N = 651) with a mean age of 20.48 years (SD = 1.98) and the majority being males (n = 390, 59.9%). It was found that the majority of participants take selfie pictures once or less per week (61.75%) and the majority uploaded their selfies less than once per week (85.56%). The social media platform used the most was WhatsApp (98.77%) although in combination with the other social media platforms.

Table 2 displays the interrelationships between self-esteem, psychological distress, problematic social media use, problematic smartphone use, selfitis, and body-self appearance. It was found that self-esteem was significantly related to problematic smartphone use, body-self appearance, and psychological distress (ps < 0.05). In addition, problematic social media use was significantly related to problematic smartphone use, selfitis, and psychological distress (ps < 0.001). Problematic smartphone use was significantly related to selfitis, and psychological distress (ps < 0.001). Body-self appearance was significantly related to selfitis and psychological distress (ps < 0.001). Furthermore, selfitis was significantly related to psychological distress (ps < 0.001). All the other interrelationships were not significant (ps > 0.05).

Table 3 displays models of the effect of problematic social media use on psychological distress with selfitis as the mediator. The study revealed that selfitis (unstandardized coefficient = 0.046; LLCI = 0.004; ULCI = 0.097) was a significant mediator in the association between problematic social media use and psychological distress. Problematic social media use directly influenced psychological distress (unstandardized coefficient of 0.542; *p* < 0.001) and selfitis (unstandardized coefficient of 0.783; SE = 0.007; *p* < 0.001). Selfitis directly influenced psychological distress (unstandardized coefficient of 0.059; p = 0.025). There was a significant total effect of problematic social media use on psychological distress (unstandardized coefficient of 0.588; *p* < 0.001).

Table 4 displays models of the effect of problematic smartphone use on psychological distress with selfitis as the mediator. The study revealed that selfitis (unstandardized coefficient = 0.040; LLCI = 0.009; ULCI = 0.081) was a significant mediator in the association between problematic social media use and psychological distress. Problematic smartphone use directly influenced psychological distress (unstandardized coefficient of 0.442; *p* < 0.001) and selfitis (unstandardized coefficient of 0.584; *p* < 0.001). Selfitis directly influenced psychological distress (unstandardized coefficient of 0.069; p = 0.008). There was a significant total effect of problematic smartphone use on psychological distress (unstandardized coefficient of 0.482; *p* < 0.001).

Table 5 displays models of the effect of body-self appearance on psychological distress with selfitis as the mediator. The study found that selfitis (unstandardized coefficient = 0.758; LLCI = 0.313; ULCI = 1.347) was a significant mediator in the association between body-self appearance and psychological distress. Body-self appearance directly influenced psychological distress (unstandardized coefficient of −4.309; *p* < 0.001) and selfitis (unstandardized coefficient of 6.381; *p* < 0.001). Selfitis directly influenced psychological distress (unstandardized coefficient of 0.119; *p* < 0.001). There was a significant total effect of body-self appearance on psychological distress (unstandardized coefficient of −3.551; *p* < 0.001).

Table 6 displays models of the effect of self-esteem on psychological distress with selfitis as the mediator. The study found that selfitis (unstandardized coefficient = 0.045; LLCI = −0.094; ULCI = 0.187) was not a significant mediator in the association between self-esteem and psychological distress. Self-esteem did not directly influence selfitis (unstandardized coefficient of 0.452; p = 0.507). Nonetheless, self-esteem directly influenced psychological distress (unstandardized coefficient of −1.937; *p* < 0.001). Selfitis directly influenced psychological distress (unstandardized coefficient of 0.100; *p* < 0.001). There was a significant total effect of self-esteem on psychological distress (unstandardized coefficient of −1.891; *p* < 0.001).

## 4. Discussion

The present study examined the mediating role of selfitis in the associations between problematic social media use and psychological distress, problematic smartphone use and psychological distress, body-self appearance and psychological distress, and self-esteem and psychological distress. The demographic characteristics indicate that majority of the participants do engage in selfie-related activities once or less per week even though almost all of them use one type of social media platform. Furthermore, the participants had above-average (or higher) self-esteem, average problematic social media use, average problematic smartphone use, better (above-average) body-self appearance, low psychological distress, and borderline level selfitis [18,69]. These results imply that the current participants seem healthy and appropriately use social media and other technological devices. Pearson’s correlation matrix indicated that the majority of the relationships were significantly positive or negative except for the relationship between self-esteem and problematic social media use, self-esteem and selfitis, problematic social media use and body-self appearance, and problematic smartphone use and body-self appearance. Self-esteem had significant negative relationships with problematic smartphone use and psychological distress as well as significant negative relationships between psychological distress and body-self appearance. This suggests that as one of these variables increases, the other decreases. The other relationships were significantly positive, indicating that as one of those variables increases, the other also increases and vice versa. The current relationships are consistent with the findings of previous studies [7,8,10,31,32,39]. It is noteworthy that the present study is the first to objectively establish a known relationship between problematic smartphone use and selfitis although there have been inferred relationships between them [24]. 

The mediating effect of selfitis in the association between problematic social media use and psychological distress was first preceded by associations between these variables. The study revealed that there were direct positive associations between these variables. This indicates that as one of the variables increases, the other variable may also increase and vice versa. This is consistent with the findings of previous studies [7,10,33,34]. In addition, there was an indirect association between problematic social media use and psychological distress via selfitis. This indicates that problematic social media use may directly influence the levels of depression, anxiety, and/or stress (psychological distress) and/or indirectly through selfie-related activities (selfitis). Therefore, young adults should be cautious with how they use social media, especially for selfie-related activities. This finding is novel as no known study has examined this mediating effect apart from one between personality type and problematic social media use [32]. It is also interesting to note that although the majority of the participants engaged in selfie-related activities at most once per week, selfitis still served as a significant pathway to psychological distress. This is especially important as previous studies reported a higher number of people who take and/or upload selfie pictures than the present study [18,21,32]. Therefore, this is an important observation as selfitis does not necessarily mean addiction and/or a mental disorder but as an assessment of selfie-related activities and maybe beyond [18,21,23]. 

Additionally, the mediating effect of selfitis in the association between problematic smartphone use and psychological distress was first preceded by associations between these variables. The study found that there were direct positive associations between these variables. This indicates that as one of the variables increases, the other variable may also increase and vice versa. This is consistent with the findings of previous studies [7,8,24,31]. Furthermore, there was an indirect association between problematic smartphone use and psychological distress via selfitis, a significant mediator. This suggests that problematic smartphone use may directly influence the levels of depression, anxiety, and/or stress (psychological distress) and/or indirectly influence them through engaging in selfie-related activities (selfitis) even if one engages in them once a week. Therefore, young adults may need to be mindful of how they use a smartphone, especially considering its multipurpose abilities so as not to affect their psychological function. This is a novel finding as no known study has examined this mediating effect apart from one between personality type and problematic social media use [32].

Furthermore, the effect of body-self appearance on psychological distress with selfitis as the mediator was first preceded by examining the association between these variables. There was a direct significant negative association between body-self appearance and psychological distress, which indicates that a better view of how a person sees, thinks, and feels about himself or herself (body-self appearance) may lead to lower levels of depression, anxiety, and/or stress (psychological distress). In addition, there were direct significant positive associations between body-self appearance and selfitis, and selfitis and psychological distress. This indicates that as a person sees, thinks, and feels better about himself or herself, there is greater likelihood that they will engage in selfie-related activities and vice versa. Moreover, the more one engages in selfie-related activities, the higher their level of depression, anxiety, and/or stress (psychological distress) may likely be and vice versa. Additionally, there was a significant indirect association between body-self appearance and psychological distress via selfitis. This suggests that body-self appearance may directly affect psychological distress or indirectly through selfitis although one may only engage in selfie-related activities once per week. That is, as long as there are selfie-related activities even once per week, selfitis becomes an important influencing factor in psychological distress. It is also important to note that selfitis changed the effect of body-self appearance on psychological distress from negative to positive. This implies that selfitis, no matter how minimal, may likely increase psychological distress. This is a novel finding. Previous studies have associated body appearance with selfie posting [19,29,45,46,47,48] and psychological distress [29,45,46]. 

In addition, the effect of self-esteem on psychological distress with selfitis as the mediator was examined by first ascertaining the associations between the variables. The study revealed that there was a significant direct negative association between self-esteem and psychological distress. In addition, there was a significant direct positive association between selfitis and psychological distress. However, there was no significant direct association between self-esteem and selfitis and there was no significant indirect association between self-esteem and psychological distress. Although there was a significant total effect of self-esteem on psychological distress, selfitis did not serve as an alternative route to the effect of self-esteem on psychological distress. Hence, an individual with low self-esteem may have higher levels of depression, anxiety, and/or stress (psychological distress) and vice versa. However, the levels of self-esteem do not have any direct influence on how the individual engages in selfie-related activities. This is contrary to previous studies which have reported that selfie posting, feedback, body satisfaction, and self-esteem are related to each other [29,45,46]. 

### Limitations

There are some limitations of this study. The participants are (fairly) young and tertiary-educated adults and hence the findings may not be generalizable to the general adult population. Future studies can recruit older and less educated adults. The study also employed a cross-sectional survey design and hence the extent of causal inference may be limited. Future research can adopt a longitudinal study to help establish causal inferences. Furthermore, self-report measures were used in the current study which may be susceptible to social desirability response bias. These limitations notwithstanding, the findings of the study can be considered reliable as robust statistical analysis was used in addition to acceptable psychometric properties. In addition, although significant mediational results were found, we did not control for the low rate of selfie-taking or uploading in our analysis. Therefore, readers should take notice of these interesting results when comparing them with other studies. Additionally, both BSMAS and SABAS had questionable reliability coefficients which may affect the reliability of the data and consequently the findings. Therefore, readers should exercise caution in overly extending the findings. Lastly, the coronavirus (COVID-19) pandemic may have influenced the results as previous studies have associated COVID-19 variables with mental health conditions [70,71,72,73,74,75]. That is, COVID-19 has been reported to have deteriorated people’s mental health via increased job stress or problematic use of social media, smartphones, and other digital devices [70,71,72,73,74,75]. 

## 5. Conclusions

The present study revealed that selfitis serves as a mediator in the associations between problematic social media use, problematic smartphone use, body-self appearance, and psychological distress but not between self-esteem and psychological distress. These findings suggest that selfitis can serve as a potential pathway by which people who overly engage in problematic social media use, problematic smartphone use, and have poor body-self appearance may experience psychological distress. Selfitis may not be a mental disorder or a behavioral addiction yet, but this study helps us to understand that selfitis extends beyond a seemingly behavioral addiction (e.g., body-self appearances). Consequently, there is a need for health communicators, school authorities, and opinion leaders to educate young adults on the adverse effects of the problematic use of technology, especially for selfitis behaviour. That is, education on better adaptive coping strategies and healthy ways of having fun should be strengthened amidst the consequences of selfie-related activities. Future studies can examine the psycho-socio-economic factors that predict selfitis behaviour among people so that clinicians and researchers are able to know which groups of people may be vulnerable to the problems related to selfitis.

## Figures and Tables

**Table 1:** Sociodemographic characteristics of participants (*N* = 651).

	Mean ± *SD* or n (%)	Missing No
Age (in years)	20.48 ± 1.98	15
Sex (males)	390 (59.9%)	3
Accommodation		49
On campus	249 (38.2%)	
Off-campus	353 (54.2%)	
Religion		33
Christian	586 (90%)	
Muslim	29 (4.5%)	
Traditional	2 (0.3%)	
Other	1 (0.2%)	
Marital status		34
Single	611 (93.9%)	
Married	4 (0.6%)	
Divorced	2 (0.3%)	
How many selfie pics do you take weekly?		
1 or fewer per week	402 (61.75%)	
2–10 per week	192 (29.49%)	
11–20 per week	37 (5.68%)	
21 or more times per week	18 (2.76%)	
How often do you upload a selfie?		
Less than once per week	557 (85.56%)	
1–5 times per week	78 (11.98%)	
6–15 times per week	5 (0.77%)	
16 or more times per week	5 (0.77%)	
Social media platforms used		
Facebook	452 (69.43%)	
Twitter	490 (75.27%)	
WhatsApp	643 (98.77%)	
Instagram	565 (86.79%)	
Snapchat	531 (81.57%)	
Tik-Tok	348 (53.46%)	

**Table 2:** Correlation matrix.

	1	2	3	4	5	6
1. Self-esteem ^a^	―					
2. Problematic Social Media Use ^b^	−0.030	―				
3. Problematic Smartphone Use ^c^	−0.088 *	0.495 **	―			
4. Body-Self Appearance ^d^	0.269 **	0.072	0.039	―		
5. Selfitis ^e^	0.026	0.236 **	0.193 **	0.178 **	―	
6. Psychological Distress ^f^	−0.162 **	0.263 **	0.236**	−0.147**	0.144 **	―
Mean	3.79	15.47	18.16	3.80	48.97	18.12
Standard Deviation	0.99	5.19	5.69	0.48	17.21	11.61

* *p* < 0.05, ** *p* < 0.001; ^a^ assessed using the single-item self-esteem scale; ^b^ assessed using the Bergen social media addiction scale; ^c^ assessed using the smartphone application-based addiction scale; ^d^ assessed using the multidimensional body-self relations questionnaire–appearance scales; ^e^ assessed using the selfitis behavior scale; ^f^ assessed using the depression anxiety stress scale-21.

**Table 3:** Models of the effect of problematic social media use on psychological distress with selfitis as the mediator.

	Unstand. Coeff.	SE or (Bootstrapping SE)	t-Value or (Bootstrapping LLCI)	*p*-Value or (Bootstrapping ULCI)
Total effect of problematic social media use on psychological distress	0.588	0.0845	6.938	<0.001
Direct effect of problematic social media use on psychological distress	0.542	0.087	6.233	<0.001
Direct effect of problematic social media use on mediator (selfitis)	0.783	0.127	6.194	<0.001
Direct effect of selfitis on psychological distress	0.059	0.026	2.240	0.025
Indirect effect of problematic social media use on psychological distress	0.046	(0.024)	(0.004)	(0.097)

Unstand. Coeff. = unstandardized coefficient; LLCI = lower limit in 95% confidence interval; ULCI = upper limit in 95% confidence interval.

**Table 4:** Models of the effect of problematic smartphone use on psychological distress with selfitis as the mediator.

	Unstand. Coeff.	SE or (Bootstrapping SE)	t-Value or (Bootstrapping LLCI)	*p*-Value or (Bootstrapping ULCI)
Total effect of smartphone addiction on psychological distress	0.482	0.078	6.192	<0.001
Direct effect of smartphone addiction on psychological distress	0.442	0.079	5.593	<0.001
Direct effect of smartphone addiction on mediator (selfitis)	0.584	0.117	5.004	<0.001
Direct effect of selfitis on psychological distress	0.069	0.026	2.651	0.008
Indirect effect of smartphone addiction on psychological distress	0.040	(0.019)	(0.009)	(0.081)

Unstand. Coeff. = unstandardized coefficient; LLCI = lower limit in 95% confidence interval; ULCI = upper limit in 95% confidence interval.

**Table 5:** Models of the effect of body-self appearance on psychological distress with selfitis as the mediator.

	Unstand. Coeff.	SE or (Bootstrapping SE)	t-Value or (Bootstrapping LLCI)	*p*-Value or (Bootstrapping ULCI)
Total effect of body-self appearance on psychological distress	−3.551	0.939	−3.783	<0.001
Direct effect of body-self appearance on psychological distress	−4.309	0.940	−4.585	<0.001
Direct effect of body-self appearance on mediator (selfitis)	6.381	1.384	4.610	<0.001
Direct effect of selfitis on psychological distress	0.119	0.026	4.527	<0.001
Indirect effect of body-self appearance on psychological distress	0.758	(0.265)	(0.313)	(1.347)

Unstand. Coeff. = unstandardized coefficient; LLCI = lower limit in 95% confidence interval; ULCI = upper limit in 95% confidence interval.

**Table 6:** Models of the effect of self-esteem on psychological distress with selfitis as the mediator.

	Unstand. Coeff.	SE or (Bootstrapping SE)	t-Value or (Bootstrapping LLCI)	*p*-Value or (Bootstrapping ULCI)
Total effect of self-esteem on psychological distress	−1.891	0.453	−4.178	<0.001
Direct effect of self-esteem on psychological distress	−1.937	0.448	−4.322	<0.001
Direct effect of self-esteem on mediator (selfitis)	0.452	0.680	0.664	0.507
Direct effect of selfitis on psychological distress	0.100	0.026	3.876	<0.001
Indirect effect of self-esteem on psychological distress	0.045	(0.070)	(−0.094)	(0.187)

Unstand. Coeff. = unstandardized coefficient; LLCI = lower limit in 95% confidence interval; ULCI = upper limit in 95% confidence interval.

## Data Availability

Data are available on reasonable request from the corresponding author.
